# Screening and Virulence of the Entomopathogenic Fungi Associated with *Chilo suppressalis* Walker

**DOI:** 10.3390/jof7010034

**Published:** 2021-01-07

**Authors:** Morteza Shahriari, Arash Zibaee, Seyyed Akbar Khodaparast, Mahmoud Fazeli-Dinan

**Affiliations:** 1 Department of Plant Protection, Faculty of Agricultural Sciences, University of Guilan, Rasht 4199613779, Iran; shahriary.uoz@gmail.com (M.S.); khodaparast@guilan.ac.ir (S.A.K.); 2 Department of Medical Entomology and Vector Control, Health Sciences Research Center, Addiction Institute, School of Public Health, Mazandaran University of Medical Sciences, Sari 4815733971, Iran; fazelidinan@gmail.com

**Keywords:** entomopathogenic fungi, *Chilo suppressalis*, isolation, identification, pathogenicity

## Abstract

The current study aimed to explore the entomopathogenic fungi associated with the larvae of *Chilo suppressalis* Walker, a serious pest of rice, in northern Iran. The collected specimens were cultured and identified through morphological and molecular methods. The 38 specimens were identified by microscopic examination and genetic sequencing of the ITS region as follows: twenty-one isolates of *Beauveria bassiana*, five isolates of *Akanthomyces lecanii,* four isolates of *Akanthomyces muscarious*, three isolates of *Metarhizium anisopliae*, two isolates *of Hirsutella subulata*, two isolates of *Trichoderma* sp. and one isolate of *Aspergillus* sp. All the identified isolates were treated on the larvae through bioassay, evaluating the amount of hydrophobin and the activities of proteases, chitinases and lipase to find their virulence. Moreover, the percentage of thermotolerant and cold activity of the isolates were tested to determine their environmental persistence. The overall results revealed the isolates of *B. bassiana,* including BBRR1, BBAL1 and BBLN1 as the most virulent and environmental adaptive isolates among the fungi associated with *C. suppressalis*.

## 1. Introduction

The rice striped stem borer, *Chilo suppressalis* Walker (Lepidoptera: Crambidae), is an economic pest of rice, annually causing significant losses in Asia, southern America and northern Africa [1]. The larvae feed intensively on rice stems and cause “whitehead” and “dead-heart” of the seedlings, which directly decrease the overall yield of rice [2]. The main control measure to suppress the *C. suppressalis* population is the wide-spraying of synthetic insecticides, including diazinon, Padan^®^ and Reagent^®^. Nevertheless, *C. suppressalis* has developed resistance to these insecticides on one hand and resulted in environmental pollution, food residuals and toxicity on non-target organisms on the other hand [3,4]. These concerns should change the management strategies of chemical insecticides toward biocontrol agents like entomopathogens. Among the entomopathogens used to manage the population of insect pests, entomopathogenic fungi cause epizootics among insect pests and appear as the prevalent natural pathogens to regulate population fluctuations of pests and subsequent losses [5]. Their presence in almost all terrestrial and aquatic ecosystems, as well as way of infection by producing different extracellular enzymes and by releasing toxic secondary metabolites, has led to the success of entomopathogenic fungi to affect noxious arthropods in agriculture, forestry and livestock [5,6].

There are many reports on the efficacy of different entomopathogenic fungi, including *Akanthomyces lecanii*, *Akanthomyces muscarious*, *Aspergillus* spp., *Beauveria bassiana*, *Isaria fumosorosea*, *Isaria sinclairii*, *Metarhizium anisopliae*, *Metarhizium rileyi*, *Nomuraea rileyi*, *Pecilomyces lilacinus* and *Purpureocillium lilacinum* against lepidopteran pests such as *Chilo suppressalis*, *Spodoptera litura*, *Spodoptera frugiperda*, *Spodoptera exigua*, *Ostrinia nubilalis*, *Helicoverpa armigera*, *Helicoverpa zea*, *Plutella xylostella*, *Duponchelia fovealis*, *Agrotis ipsilon*, *Pieris rapae*, *Trichoplusia ni*, *Ocinara varians*, *Galleria mellonella*, *Plodia interpunctella*, *Ephestia kuehniella* [7,8,9,10,11,12,13,14,15,16,17,18,19]. These agents have generally shown to be safe for humans with the least effects on non-targets while they are relatively sensitive to environmental conditions, mainly heat, cold and UV radiation, so it is imperative to find isolates adaptable to these constraints for formulation and field application [20,21,22,23].

Exogenous isolates of the entomopathogenic fungi that were commercialized as pest biocontrol agents in different countries may be ineffective on some pests due to environmental suitability and strain differences related to the host [24]. Therefore, the application of local isolates may be a promising choice mainly in case of ecological suitability with pest species and lower hazards on non-target organisms compared to exotic strains [22,25,26,27]. These points were verified by several studies that demonstrate the virulence of isolates belonging to the same fungal species could be different because of genetic variations occurring in a specific geographical distribution [28,29,30]. The provinces of Guilan and Mazandaran are located in the north of Iran with high humidity, moderate annual temperatures and heavy rainfall, in which these conditions are appropriate for entomopathogenic fungi [5]. The rice fields of northern Iran, known as a reservoir of *C. suppressalis* [31], can represent ideal sites to study the existence of entomopathogenic fungi with natural enzootics to *C. suppressalis*, so the aims of our study were to; (a) isolate and identify different entomopathogenic fungi from fungus-infected *C. suppressalis* larvae, (b) evaluate the virulence of these fungi against the larvae of *C. suppressalis*, (c) examine the infection process of these isolates by the production of extracellular secretions and (d) compare the conidial germination of the fungal isolates after exposure to heat and cold.

## 2. Materials and Methods

### 2.1. Collection and Morphological Identification

The collection sites were all the municipal regions of Guilan and Mazandaran provinces in the north of Iran (Mazandaran and Guilan, Iran) with the highly cultivated area of rice. In each site, the remained stems of rice within the paddy fields were opened, and the infected larvae of *C. suppressalis* were collected and kept in sterile centrifuge tubes. The infected larvae were recognized according to the mycelial growth outside the larval body. Once the samples were transferred to the laboratory, the larvae were surface disinfected with sodium hypochlorite (2%) for 3 min and rinsed three times in sterile distilled water [27]. The larvae were then transferred on potato dextrose agar (PDA, Merck) plates and incubated at 25 °C for 2–4 days for fungal development. Afterward, the fungal mycelia were picked up and transferred to fresh PDA plates for purification. Finally, single-spore cultures were gathered according to the method described by Fang [32] and cultured on PDA slants. All collected specimens were inoculated on PDA plates and incubated at 25 °C in the dark for 14 days. For microscopic examination, mycelia and conidia from fungal specimens were mounted on a sliding glass and observed at 100× magnification on a phase-contrast microscope (Canon INC DS126311, Taiwan). Morphological identification of the specimens was made based on conidial morphology, shape, color and size based on the following literature: *Akanthomyces* spp. isolates [28,33,34], *Beauveria* spp. Isolates [28,34,35], *Hirsutella* spp. isolates [34,36,37,38] and *Metarhizium* spp. isolates [34,39].

### 2.2. Genomic DNA Extraction and PCR

DNA extraction was done using the protocol of Montero-Pau et al. [40]. Briefly, the mass mycelia of the specimen grown in PDA media were transferred to the 1.5 mL tubes containing 100 μL of alkaline lysis buffer (0.2 mM disodium ethylene diamide tetraacetic acid, 25 mM NaOH, pH 8.0, Merck) and centrifuged for 30 min at 2000× *g*. Then, the tubes were incubated at 95 °C for 30 min and cooled on ice for five min. Finally, 100 μL of Tris-HCl solution (Sigma-Aldrich, Vienna, Austria; 40 mM, pH 5.0) was added to the tubes, vortexed and maintained at −20 °C. The extracted solution was used as a template for PCR.

To amplify the internal transcribed spacers (ITS5-5.8S-ITS4), ITS5 (5′GGAAGTAAAAGTCGTAACAAGG3′) and ITS4 (5′-TCCTCCGCTTATTGATATGC-3′) primers were synthesized as previously described [41]. The PCR reaction mixture consisted of 12.5 μL of master mix (Including 10× PCR buffer. MgCl2, dNTPS TaqPolymerase, CinnaClone, Tehran, Iran), 7.5 μL of double-distilled H_2_O, 1 μL of each primer and 3 μL of DNA solution. PCR was carried out using a thermal cycler (Eppendorf Personal, Darmstadt, Germany) with the following reaction parameters: an initial denaturation for 2 min at 94 °C, 30 cycles of 94 °C for 30 s, 53 °C for 30 s and 72 °C for 1 min and a final extension for 5 min at 72 °C. Amplified PCR products were visualized by electrophoresis on 1% agarose gels. The PCR products were sent to a sequencing service company (Royan Zistagene Co., Tehran, Iran) for purification and sequencing. Finally, sequences were compared with other fungi using the BLAST search tool in NCBI (https://blast.ncbi.nlm.nih.gov/Blast.cgi).

### 2.3. Insect Rearing

The stock population of *C. suppressalis* was established by collecting the egg patches from rice fields of Amol, northern Iran. The eggs were kept in a growth chamber at 25 ± 2 °C, 85 ± 5% R.H. and 16:8 (L:D) h of photoperiod. The newly hatched larvae were transferred to the tubes (20 × 15 cm) supplied by rice seedlings of Hashemi variety. Quality of food was monitored every day, and the old cutting stems were replaced by the fresh ones [42]. Rearing was continued for three generations at the same controlled conditions.

### 2.4. Bioassay

Conidia of the two-week-old PDA cultures of the identified isolates were removed by a scalpel then suspended in sterile distilled water containing Tween-80 (0.02%). The concentrations of 10^2^ to 10^8^ conidia/mL from each isolate were separately prepared based on the preliminary tests. Early fourth instar larvae of *C. suppressalis* were randomly selected and separately dipped in the serial concentrations of each isolate while the control larvae were dipped in an aqueous solution of 0.02% Tween-80 alone. The bioassays were done in three replicates with ten larvae per replication, and the larvae were maintained at the rearing condition for the whole bioassay period. Mortality was recorded within 7 days, and LC_50_ values were determined using POLO-Plus software. For calculation of LT_50_, mortality was recorded until the death of all larvae at 10^8^ conidia/mL concentration.

### 2.5. Hydrophobin Protein Extraction and Estimation

Hydrophobin content was determined according to the method described by Ying and Feng [43]. Conidia from the two-week-old cultures were added to 1 mL of 2% SDS aqueous solution containing β-mercaptoethanol (5%, Merck) and incubated in a boiling water bath for 10 min before being centrifuged at 22,000× *g* and 4 °C for 10 min. The supernatant was removed, and conidia were rinsed twice in distilled water to eliminate SDS (Merck) soluble proteins. Samples were incubated in 1 mL formic acid at zero temperature for 2 h before being centrifuged at 22,000× *g* and 4 °C for 10 min. The supernatant was transferred into fresh tubes, and 0.5 mL of distilled water was added to the samples. Afterward, 0.75 mL of 45% NaOH solution added to the mixture and maintained at 4 °C overnight. The proteins were separated from the supernatant by centrifugation at 22,000× *g* and 4 °C for 10 min. The extractable proteins of formic acid were rinsed twice with ethanol solution (3:1, *v*/*v*) and then dissolved in 2% SDS to quantitatively determine the amount of protein as mg/mL of conidia using the procedure of Lowry et al. [44].

### 2.6. Liquid Culture for Enzyme Production of the Isolates

The liquid media used for biochemical production of the extracellular enzymes contained; 0.02% of KH_2_PO_4_; 0.01% of CaCl_2_; 0.01% of MgSO_4_; 0.02% of Na_2_HPO_4_; 0.01% of ZnCl_2_ and 0.01% of yeast extract (Merck). The media were inoculated with 1 mL of 10^8^ conidia/mL concentration of each isolate separately and 5% (weight) of larval cuticle was added to each flask containing liquid media. Then the flasks were kept on a rotatory shaker (70 rev/min) for 8 days at 25 ± 1 °C [3].

### 2.7. Sample Preparations for Enzymatic Assays

After 8 days, the culture flasks were harvested by centrifugation at 10,000× *g* for 30 min and washed in ice-cold Tris-HCl (25 mM, pH 8). Weighed mycelia were ground to a fine powder, suspended in DW, homogenized and centrifuged at 22,000× *g* and 4 °C for 30 min to obtain the supernatant of enzyme assay [12].

#### 2.7.1. Assay of Proteases

Activities of subtilisin-like (Pr1) and trypsin-like (Pr2) as the two key fungal proteases were determined by the specific substrates of succinyl-(alanine) 2-prolinephenylalanine-*p*-nitroanilide and benzoylphenylalanine-valine-arginine-*p*-nitroanilide (Sigma-Aldrich, Co., Vienna, Austria), respectively. The reaction mixture contained 100 µL of Tris-HCl buffer (20 mM, pH 8), 30 µL of each substrate separately and 20 µL of enzyme solution. The mixture was incubated at 25 °C for 10 min, then 100 μL of trichloroacetic acid (TCA, 30%) was added, and the absorbance was recorded at 405 nm [3].

#### 2.7.2. Lipase Assay

Lipase assay was done using the method of Tsujita et al. [45]. Fifty microliters of *p*-nitrophenyl butyrate (27 mM, Sigma-Aldrich, Co., Vienna, Austria) as substrate, 20 μL enzyme solution and 100 μL of Tris-HCl buffer (20 mM, pH 7) were incorporated and incubated at 37 °C for 5 min. Then, 100 μL of NaOH (1 N) was added to each tube, and the absorbance was recorded at 405 nm.

#### 2.7.3. Endochitinase Assay

Twenty microliters of the enzyme solution were added to 50 μL of 0.5% colloidal chitin (Sigma-Aldrich, Co., Vienna, Austria) as substrate and 100 μL of Tris-HCl buffer (20 mM, pH 7). Then, the samples were incubated in a water bath of 30 °C for 60 min. Then, 100 μL of dinitrosalisylic acid (DNS, Sigma-Aldrich, Vienna, Austria) was added, the incubation was prolonged for 10 min at boiling water, and the absorbance was recorded at 545 nm [46].

#### 2.7.4. Exochitinase Assay

The activity of exochitinase was assayed by 200 μL of *p*-nitrophenyl-N-acetyl-β-D-glucosaminide (pNPg; Sigma-Aldrich, Co., Vienna, Austria) solution (1 mg pNPg per mL of distilled water) as substrate, 25 μL of enzyme solution and 500 μL of Tris-HCl (25 mM, pH 7) which was incubated at 40 °C for 20 h. Then, the mixture was centrifuged at 13,000 rpm at 4 °C, and the supernatant was added to 200 μL of sodium tetraborate-NaOH buffer (125 mM, pH 10) before to read the absorbance at 400 nm. The extinction coefficient of 18.5 Mm^−1^ −cm^−1^ was considered for activity calculation based on the following formula:

Volume activity (U/mL) = [ΔOD (OD test–OD blank) × V_t_ × df]/(18.5 × t × 1.0 × Vs) where, V_t_ = total volume; Vs = sample volume; 18.5 = millimolar extinction coefficient of *p*-nitrophenol under the assay condition; 1.0 = lightpath length (cm); t = reaction time; and df = dilution factor [46].

### 2.8. Protein Assay

The method of Lowry et al. [44] was used to determine the content of protein in the provided samples. Twenty microliters of the enzyme solution were added into 100 µL of reagent (Ziest Chem. Co., Tehran, Iran) and incubated for 30 min before reading the absorbance at 545 nm.

### 2.9. Effects of Thermotolerance and Cold Activity on Conidial Germination

To measure thermotolerance for conidial germination, 100 μL of conidial suspensions (5 × 10^6^ conidia/mL) from each isolate was transferred to 1.5 mL tubes and kept in a thermal cycler adjusted to 45 °C. After 1 h and 2 h, 20 μL of conidial suspensions were removed and plated (without spreading) on PDA. Finally, plates were maintained at 25 °C, and conidial germination was counted after 24 h by microscopic observation. Moreover, 20 μL of a conidial suspension (5 × 106 conidia/mL) was plated (without spreading) on PDA and kept at 5 °C to determine the germination after 7 and 14 days in cold condition. In both experiments, the conidial control suspensions were inoculated on PDA at 25 °C. The relative percent germination was estimated by comparing conidial germination to untreated isolates, and at least 100 conidia were counted for each treatment in every test [27].

### 2.10. Statistical Analysis

Probit analysis was done to determine LC_50_ and LT_50_ values at the corresponding 95% confidence interval (CI) values by using POLO-Plus software. Biochemical data and germination of conidia were compared by one-way analysis of variance (ANOVA) followed by Tukey’s test. Differences among control and treatments were statistically considered at a probability of less than 5% and marked by different letters.

## 3. Results and Discussion

### 3.1. Screening and Identification of Fungi

**Figure 1:**
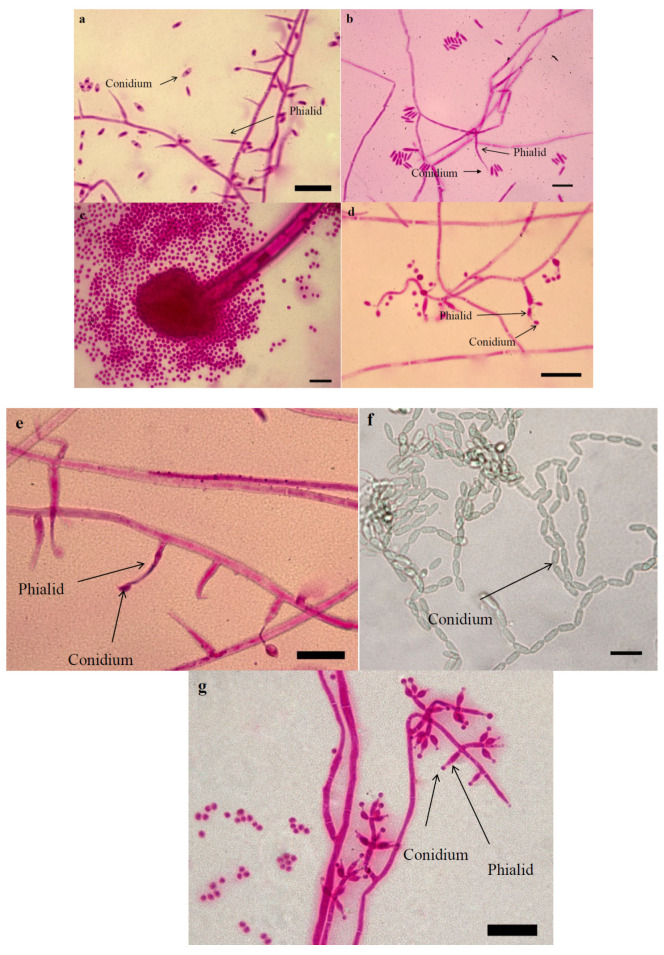
Morphological characteristics (conidium and phialid) of the fungus associated with the larvae of *Chilo suppressalis*. (**a**) *Akanthomyces lecanii*, (**b**) *Akanthomyces muscarius*, (**c**) *Aspergillus* sp., (**d**) *Beauveria bassiana*, (**e**) *Hirutella subulate*, (**f**) *Metarhizium anisopliae* complex, (**g**) *Trichoderma* sp. Bars are 20 micrometers.

A total of 38 fungal specimens were collected from *C. suppressalis* larvae, which were naturally infected by fungi in the rice fields of northern Iran. The specimens were morphologically identified as *Akanthomyces lecanii* (×5 isolates), *Akanthomyces muscarius* (×4 isolates), *Aspergillus* sp. (×1 isolate), *Beauveria bassiana* (×21 isolates), *Hirutella subulata* (×2 isolates), *Metarhizium anisopliae* complex (×3 isolates) and *Trichoderma* sp. (×2 isolates) (Table 1, Figure 1). Among all specimens, it was the first report of the natural occurrence of *H. subulata* in Iran. In *A. lecanii*, conidiogenous cells were phialidic, phialides approximately small, length size of 11–16 μm and width size of 1.4–2 μm, aculeate and strongly tapering, solitary or in whorls 3–6, conidial shape ellipsoidal-cylindrical, length size 4.1–5.2 μm and width size of 1.3–2.1 μm (Table 1, Figure 1a). In *A. muscarious*, conidiogenous cells were phialidic; phialides burned straight on prostrate hyphae or on secondary branches, phialides generally tall and slender and longer than *A. lecanii*, length size of 28–35 μm and width size of 1.6–2 μm, conidial shape cylindrical and longer than *A. lecanii*, length size 6.5–9.5 μm and width size of 1.5–1.9 μm (Table 1, Figure 1b). The major difference between the species of *Akanthomyces* is the shape and size of phialides that our specimens matched accurately match the description given by Zare and Gams [33]. In *Aspergillus* sp. conidial shape was globose, length size 1.8–2.3 μm and width size of 1.8–2.3 μm (Table 1, Figure 1c). In addition, the Conidial shape of *B. bassiana* isolates was globose with a length size of 2.1–3.2 μm and width size of 2–3 μm; conidiogenous cells were phialidic; the phialides were flask-shaped, swollen at the base or near the base and tapering at the apex. In addition, the conidiogenous cells were usually solitary or in a cluster of up to five (Table 1, Figure 1d), which appeared typical of those described by other researchers [28,34,35]. The major difference between the species of *Beauveria* is the shape and size of conidia [28]. In *H. subulata*, conidiogenous cells were phialidic, phialides scattered, and the lower phialides were narrow ellipsoid; the conidial shape was ovoid and in a chain, length size 5.5–6.9 μm and width size of 3.9–5.1 μm (Table 1, Figure 1e). Our isolates were compared with Yoon et al. [37], and significant differences were not observed among them. The conidial shape of *M. anisopliae* isolates was oblong oval with a length size of 6.8–7.8 μm and a width size of 2.6–3.7 μm (Table 1, Figure 1f). Conidia were the only morphological particular that reliably distinguishes several *Metarhizium* species [39]. In *Trichoderma* sp. conidial shape was globose, length size 2.2–3.1 μm and width size of 2.1–2.9 μm (Table 1, Figure 1g).

The ITS5-5.8S-ITS4 region was used for molecular analysis, confirming identifications of the fungal isolates. The amplified ITS region from all specimens showed approximately 600 bp-sized fragments, and the samples were sequenced and compared in the GenBank database. Results of the ITS sequence data were consistent with those obtained using morphological studies. After submission to the GenBank database, the fungal isolates were renamed based on the given code (Table 1). In the current study, *B. bassiana* was the most frequent detected fungus in rice collected from fields similar to others studies that have shown *B. bassiana* as the most widespread entomopathogenic fungi in the endemic Moroccan forests of *Argania spinosa*, Switzerland, Spain, China and Southern Italy [22,47,48,49,50]. Moreover, *B. bassiana* has been reported as the major pathogen of insects in more than 200 species that have been identified from the soil and dead insects in nature [24,25,51,52].

### 3.2. Bioassay

Only the isolates of *B. bassiana*, *M. anisopliae and H. subulata* caused mortality against the larvae of *C. suppressalis*. A comparison of LC_50_ and LT_50_ values indicated the significant differences among the isolates. The most virulent, isolate BBLN1 (1 × 10^4^ conidia/mL), had the least LC_50_ value, followed by BBAL1 (2.1 × 10^4^ conidia/mL), BBRR1 (2.2 × 10^4^ conidia/mL), BBLN2 (5.4 × 10^4^ conidia/mL), BBAL3 (5.6 × 10^4^ conidia/mL) and MASA (7.1 × 10^4^ conidia/mL), while HSBL (1.6 × 10^6^ conidia/mL), HSAL (7.9 × 10^5^ conidia/mL) and BBLD5 (4.4 × 10^5^ conidia/mL) showed the comparatively high LC_50_ values (Table 2). Moreover, the least LT_50_ values were obtained to be 2.71, 3.15, 3.45, 3.66 and 3.69 days for the larvae treated by BBRR1, BBLN1, BBAL1, BBAL4 and MASA, respectively (Table 3). These results revealed that BBRR1, BBLN1 and BBAL1 isolates of *B. bassiana* had higher efficacy than the other isolates on *C. suppressalis* larvae with a lesser concentration of conidia with a shorter time (days) to kill 50% of the larval population. Jandricic et al. [53] reported the higher virulence of *B. bassiana* isolates against the *Myzus persicae* Sulzer, *Aphis gossypii* Glover and *Aulacorthum solani* Kaltenbach (Hemiptera: Aphididae) compared to *M. anisopliae* isolates. Ramzi and Zibaee [12] showed that the two commercial isolates of *B. bassiana* and *B. bassiana* (BB1 and BB2) had the higher virulence against *C. suppressalis* larvae compared to *A. lecanii*, *I. fumosoroseus and M. anisopliae*. In addition, the higher virulence of the different isolates of *B. bassiana* and *M. anisopliae* has been observed on the boll weevil *Anthonomus grandis* Boheman (Coleoptera: Curculionidae) [29]. In our study, the least virulence of HSAL and HSBL as the two isolates of *H. subulata* were obtained compared to *B. bassiana* and *M. anisopliae* isolates, which may be correlated with low germination and sporulation rates in addition to the low activities of the extracellular enzymes of these isolates (see below) [54]. Finally, the isolates of *A. lecanii* and *A. muscarious* showed no mortality against *C. suppressalis* larvae. This case may be attributed to host–pathogen interaction between these isolates and the larvae of *C. suppressalis,* such as efficient attachment of conidia to the integument, negative impacts of integument composition with penetration tube of the fungi and immune responses of the larvae toward conidia. All these phenomena deserve detailed experiments to precisely elucidate the case.

### 3.3. Hydrophobin

The highest amounts of hydrophobin recorded in BBAL1, BBLD5, BBLD1, BBSI, BBBL1, BBLN1, BBLN2, HSAL and MASA, respectively (Table 4), while the least amounts of hydrophobin were in TSRT, ASAI and TSAH (Table 4). Entomopathogenic fungi achieve the nutrients at host bodies through the cuticle, so the first step of pathogenesis is adhesion to the integument. Therefore, the external surface of conidia has a fundamental protein with a hydrophobic rodlet layer that connects to the insect epicuticle [6]. Hydrophobins are a class of unique fungal proteins important in sporulation, pathogenesis, thermotolerant, growth and development of fungi [43,55,56]. Some studies reported that inhibition of hydrophobin gene expression negatively affected pigmentation, conidiation, hydrophobicity and virulence of entomopathogenic fungi [24,55,57]. Our findings revealed that the lesser amount of hydrophobin could be one of the reasons for no mortality of some isolates against the larvae of *C. suppressalis*. In fact, the proper attachment of the conidia to the host cuticle and subsequent germinations are the primarily important steps to effective infection by entomopathogenic fungi. The higher amounts of hydrophobin were obtained in the isolates with the more virulence-like BBLN1 and BBLN2. Although the higher amounts of hydrophobin were also found in HSAL with the least virulence, it should be noted that hydrophobin is not necessarily the main factor in the virulence of a fungus, but it only shows the better interaction with the host cuticle.

### 3.4. Extracellular Enzymes

**Figure 2:**
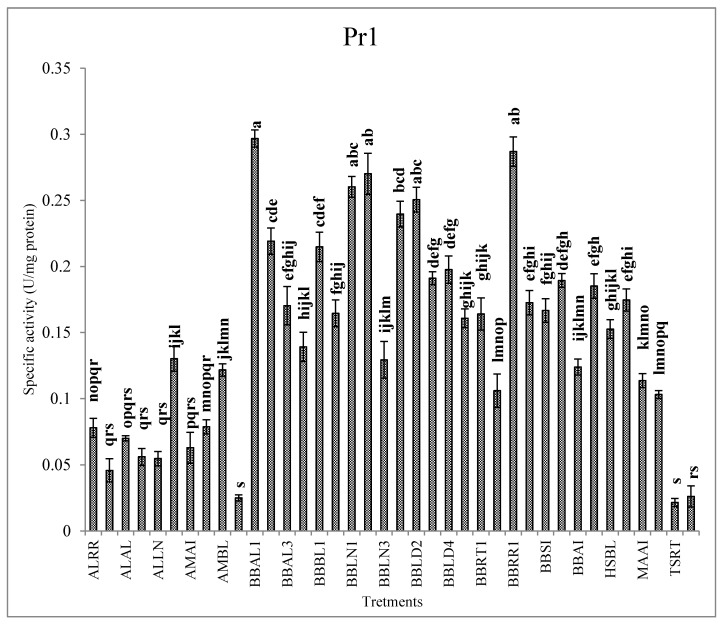

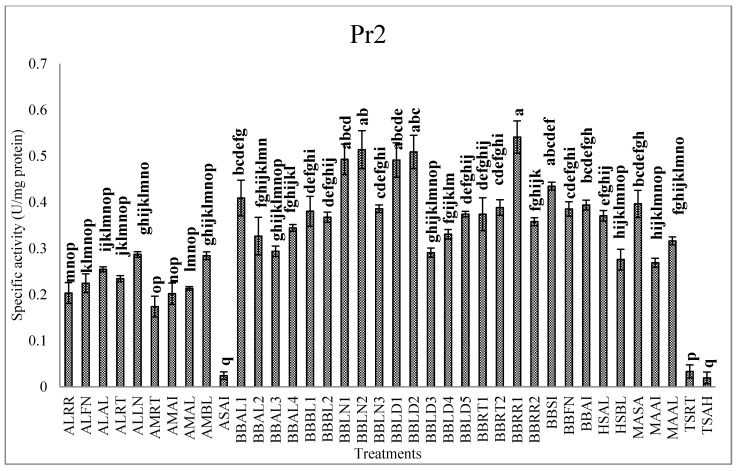
Activities of the proteases (U/mg protein, Mean ± SE) in the liquid culture media of the entomopathogenic fungi in the presence of *C. suppressalis* cuticle. Statistical differences are shown by different letters (Tukey’s test, *p* ≤ 0.05).

**Figure 3:**
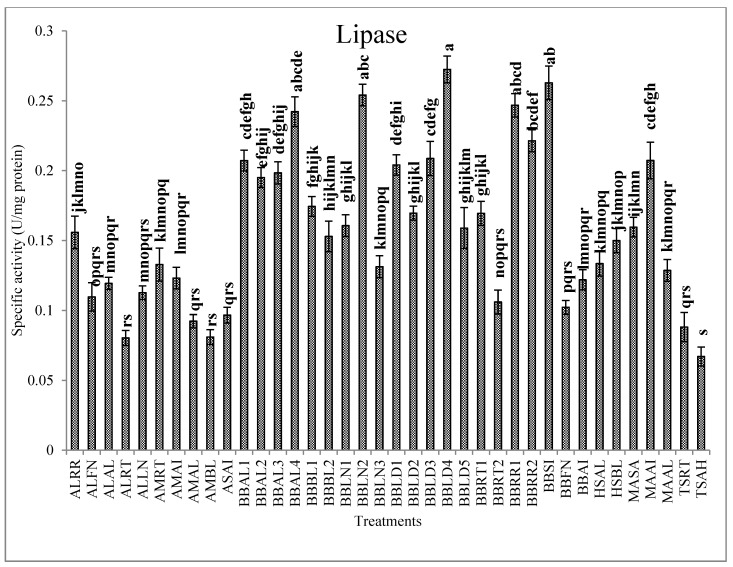
Activity of the lipase (U/mg protein, Mean ± SE) in the liquid culture media of the entomopathogenic fungi in the presence of *C. suppressalis* cuticle. Statistical differences are shown by different letters (Tukey’s test, *p* ≤ 0.05).

**Figure 4:**
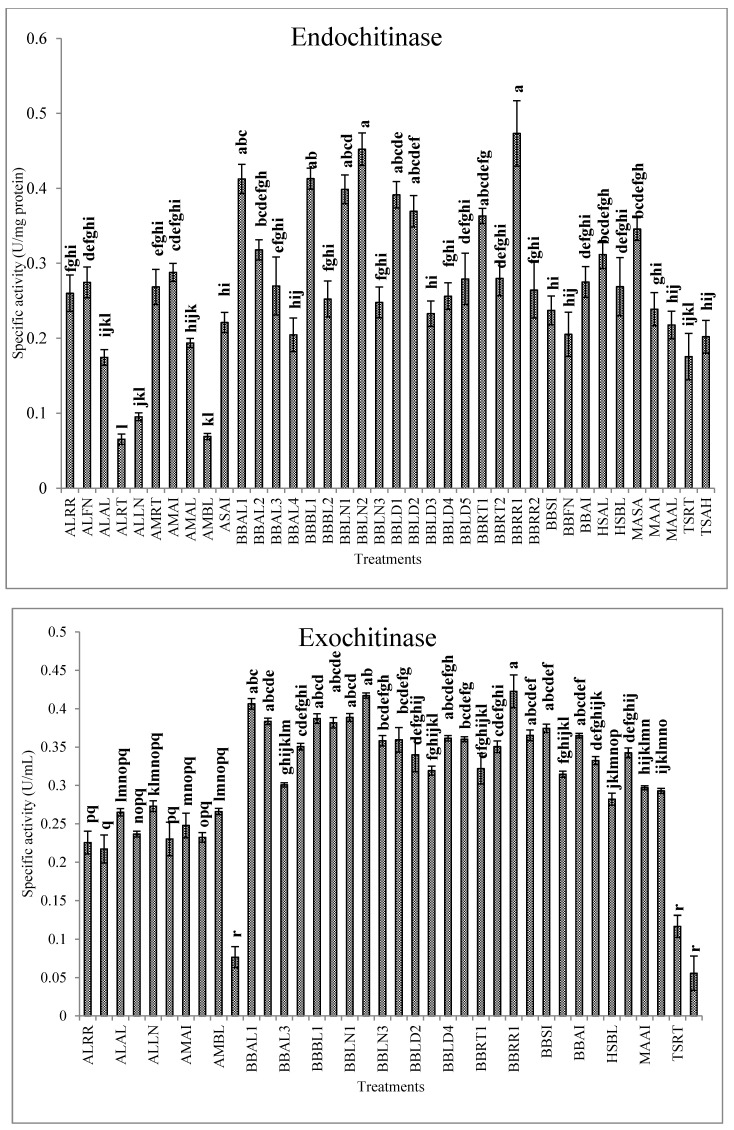
Activities of the chitinases (U/mg protein, Mean ± SE) in the liquid culture media of the entomopathogenic fungi in the presence of *C. suppressalis* cuticle. Statistical differences are shown by different letters (Tukey’s test, *p* ≤ 0.05).

The conidia of entomopathogenic fungi attach to the cuticle of host insects, germinate and penetrate to the hemocoel with the assistance of extracellular enzymes, such as chitinases, proteases and lipases [58]. Trypsin (Pr1) and subtilisin-like (Pr2) proteases are the primitive synthesized enzymes to simplify penetration of the hyphae into the host body. Then, synthesis of the chitinases increases the penetration efficiency [59], and finally, lipases involved in hydrolyzing lipid derivatives within the cuticle and facilitating the infection of host cells [60]. Our results revealed differences in the activities of extracellular enzymes between the fungal isolates. Isolates BBAL1, BBRR1, BBLN2, BBLN1 and BBLD2 demonstrated the highest activity of Pr1 while the least activity was observed in ASAI, TSRT and TSAH isolates (Figure 2). In the case of Pr2, BBRR1, BBLN2, BBLD2 and BBLN1, isolates showed the highest activity (Figure 2). The highest activity of lipase was recorded in BBLD4, BBSI, BBLN2 and BBRR1 isolates (Figure 3). BBRR1, BBLN2, BBAL1 and BBLN1 isolates showed the highest activity of exochitinase (Figure 4). In the case of endochitinase, the highest activity was obtained in BBRR1, BBLN2, BBBL1, BBAL1 and BBLN1 isolates (Figure 4). The higher Pr1 activity in the given isolates indicates the capability of protein digestion by these isolates in the initial stages of infection, so the efficiency of this enzyme may ensure the success of other enzymes to feasible penetration through insect cuticle. Charnley and St. Leger [61] believe in facilitating the cuticle infiltration by the proteases produced during invasion prior to chitinases during later steps. They concluded the major role of proteases in cuticle penetration compared to chitinases. Ramzi and Zibaee [12] demonstrated the different levels of proteinases, chitinase and lipase produced by *B. bassiana*, *M. anisopliae*, *L. lecanii* and *I. fumosoroseus* in the larvae *C. suppressalis* in which the isolates with the highest enzymatic activity led to the higher mortality Lu et al. [1] showed, the higher levels of protease and chitinase produced by ZJLSP09 isolate of *Lecanicilium* sp. in comparison with ZJLA07 and ZJLP08 isolates which were related to mortality in *Diaphorina citri* Kuwayana (Hemiptera: Psyllidae). Maqsoudi et al. [62] reported that the isolate of *B. bassiana* with the higher activity of proteases and chitinases led to the lower LC50 and LT50 values against *Pseudococcus viburni* Signoret (Hemiptera: Pseudococcidae). In our study, no clear correlation was obtained between lipase production and virulence of isolates, similar to earlier studies [12,23,63,64]. This conclusion on lipase may be more obvious in the case of BBLN2, which is the only isolate with higher virulence and lipase activity. Other isolates with higher virulence showed lower lipase activity. It seems that lipases are more important in the utilization of integument lipids for fungal development, not necessarily penetration. In contrast, the isolates with the higher virulence demonstrated the higher activity of proteases and chitinases, mainly BBLN1, BBLN2 and BBRR1. These findings apparently disclosure the correlation between efficiency of extracellular enzymes and higher virulence of the entomopathogenic fungi. Such isolates properly or rapidly penetrate through host cuticle with efficient cleavage of polypeptide and carbohydrate bonds then achieve hemocoel to continue the latter steps of infection. It should be mentioned that this process is accompanied by better production of blastospores and secondary metabolites within the host hemocoel to impose virulence on infected individuals.

### 3.5. Effects of Thermotolerance and Cold Activity on Conidial Germination

The inactivation and delay of conidia germination caused by heat and cold as the most important environmental factors reduce the efficiency of the entomopathogenic fungi as the biocontrol agents from both the virulence against host and persistence in ecosystems. Selection of the entomopathogenic fungi that tolerate thermal fluctuations te is necessary before field application [21,27,65]. The effect of thermotolerance on the germination rate of the conidia of the collected isolates in the current study have shown in Table 5. Fifteen isolates demonstrated a germination rate of more than 50% after 1 h exposure to 45 °C, while only one isolate exhibited a high tolerance after 2 h (Table 5). After 2 h of exposure, the isolate thermotolerance could be divided into three classes: low (below 30%), moderate (between 30% and 60%) and high tolerance (above 60%). Among the isolates, *Aspergillus* sp. (ASAI) showed high tolerance. Moderate tolerances were observed in four isolates of *B. bassiana* (BBLN1, BBAL1, BBLN2 and BBLD2), three isolates of *M. anisopliae* (MASA, MAAI and MAAL) and two isolates of *Trichoderma* sp. (TSAH and TSRT). The other isolates were low tolerance to the heat of 45 °C. Similar results have been reported by Lee et al. [27] as the rate of the conidial germination in *B. bassiana*, *M. anisopliae* and *Lecanicillium attenuatum* significantly reduced after 2 h at 45 °C. Rivas et al. [66] demonstrated the significant lower conidial germination of *Lecanicillium* isolates after incubation at 32 °C. The susceptibility of *Metarhizium* isolates to high temperatures (45 °C) was demonstrated by Rangel et al. [65]. Exposure to 35 °C for 10 min harmed the conidial germination of *B. bassiana,* but the *M. anisopliae* isolate germinated readily at this temperature [67]. Generally, the optimal temperature for conidia germination and growth of entomopathogenic fungi is between 23 and 28 °C. The growth was reduced above 30 °C, and it was totally inhibited above 34 °C [20,27,65,66,67]. Our results imply only *Aspergillus* sp. isolates as the highly thermotolerant isolate has although it had no virulence against the larvae of *C. suppressalis*. Finally, the cold activity of fungal isolates was examined through the treatment of the conidia at 5 °C for one and two weeks. All the isolates showed high activity (above 80%) at 5 °C for both time intervals except for *Aspergillus* sp. (ASAI) (Table 5). Lee et al. [27] reported the high cold activity at 7–14 days for almost all collected isolates. Based on earlier reports, most entomopathogenic fungi have high cold activity, although germination and sporulation may be delayed or stopped at a cold temperature [21,27,68,69]. Such a property may be important in the survival of entomopathogenic fungi in cold periods of the year.

## 4. Conclusions

Despite there are several commercial mycoinsecticide against major insect pests of agricultural products, exploration to native entomopathogenic fungi in each region may contribute to represent isolates or species with environmental and host adaptations. These adaptations may ensure virulence, environmental persistence and the least non-target effects of native isolates when they are used in field scale. The overall results of our study revealed the isolates of *B. bassiana,* including BBRR1, BBAL1 and BBLN1, were the most virulent and environmental adaptive isolates among the fungi associated with *C. suppressalis* based on bioassay, biochemical traits and thermal experiments. These isolates should undergo further studies considering field trials on the target pest and some predators and parasitoids of rice fields to better elucidate their role in pathogenesis.

## Figures and Tables

**Table 1:** Morphological characteristics and GenBank accession number of the collected fungi from the larvae of *Chilo suppressalis*.

Identification	Isolates	Conidia Size (um)	Shape of Conidia	Color of Conidia or Colony	Gene Bank Accession No.	Ident (%)
*Akanthomyces lecanii*	ALRR	4.3 ± 0.07 × 1.9 ± 0.04	Ellipsoidal-Cylindrical	White	MW143527	99.44
	ALFN	4.9 ± 0.1 × 1.7 ± 0.04	Ellipsoidal-Cylindrical	White	MW143528	99.08
	ALAL	4.5 ± 0.06 × 1.8 ± 0.04	Ellipsoidal-Cylindrical	White	MW143529	99.08
	ALRT	4.8 ± 0.06 × 1.6 ± 0.05	Ellipsoidal-Cylindrical	White	MW143531	99.44
	ALLN	4.5 ± 0.07 × 1.5 ± 0.03	Ellipsoidal-Cylindrical	White	MW143530	99.44
*Akanthomyces muscarius*	AMRT	8 ± 0.09 × 1.7 ± 0.04	Cylindrical	White	MW143523	99.82
	AMAI	7.8 ± 0.08 × 1.8 ± 0.03	Cylindrical	White	MW143524	98.40
	AMAL	8.2 ± 0.04 × 1.8 ± 0.05	Cylindrical	White	MW143525	99.80
	AMBL	7.5 ± 0.02 × 1.9 ± 0.06	Cylindrical	White	MW143526	99.61
*Aspergillus* sp.	ASAI	2 ± 0.05 × 2 ± 0.04	Globose	Dark green	MW143532	99.82
*Beauveria bassiana*	BBAL1	2.7 ± 0.07 × 2.5 ± 0.07	Globose	White	MW143537	99.81
	BBAL2	2.8 ± 0.07 × 2.5 ± 0.05	Globose	White	MW143538	100
	BBAL3	3 ± 0.05 × 2.8 ± 0.09	Globose	White	MW143539	99.81
	BBAL4	2.9 ± 0.04 × 2.8 ± 0.08	Globose	White	MW143540	100
	BBBL1	2.9 ± 0.06 × 2.6 ± 0.06	Globose	White	MW143541	99.61
	BBBL2	3.3 ± 0.05 × 2.9 ± 0.09	Globose	White	MW143542	99.61
	BBLN1	2.8 ± 0.08 × 2.6 ± 0.08	Globose	White	MW143546	99.63
	BBLN2	2.9 ± 0.05 × 2.8 ± 0.07	Globose	White	MW143547	99.81
	BBLN3	2.5 ± 0.06 × 2.4 ± 0.04	Globose	White	MW143548	99.63
	BBLD1	2.8 ± 0.05 × 2.6 ± 0.05	Globose	White	MW143549	99.43
	BBLD2	2.9 ± 0.04 × 2.7 ± 0.07	Globose	White	MW143550	99.44
	BBLD3	2.6 ± 0.08 × 2.4 ± 0.05	Globose	White	MW143551	99.26
	BBLD4	2.9 ± 0.04 × 2.5 ± 0.06	Globose	White	MW143552	99.26
	BBLD5	2.8 ± 0.05 × 2.6 ± 0.09	Globose	White	MW143553	99.81
	BBRT1	2.9 ± 0.05 × 2.7 ± 0.09	Globose	White	MW143533	100
	BBRT2	2.8 ± 0.04 × 2.7 ± 0.04	Globose	White	MW143534	100
	BBRR1	2.7 ± 0.08 × 2.4 ± 0.06	Globose	White	MW143535	100
	BBRR2	2.9 ± 0.08 × 2.7 ± 0.07	Globose	White	MW143536	100
	BBSI	2.9 ± 0.06 × 2.7 ± 0.07	Globose	White	MW143544	98.90
	BBFN	2.9 ± 0.04 × 2.7 ± 0.08	Globose	White	MW143543	100
	BBAI	3 ± 0.06 × 2.9 ± 0.08	Globose	White	MW143545	100
*Hirutella subulata*	HSAL	5.9 ± 0.1 × 4.5 ± 0.06	Ovoid	White-cream	MW143559	99.61
	HSBL	6 ± 0.2 × 4.7 ± 0.08	Ovoid	White-cream	MW143560	99.61
*Metarhizium anisopliae* complex	MASA	7.6 ± 0.1 × 3.2 ± 0.07	Oblong oval	Brown-green	MW143556	100
	MAAI	7.4 ± 0.8 × 3.3 ± 0.05	Oblong oval	Brown-green	MW143557	99.81
	MAAL	7.7 ± 0.4 × 3.6 ± 0.05	Oblong oval	Brown-green	MW143558	99.81
*Trichoderma* sp	TSRT	2.7 ± 0.08 × 2.7 ± 0.09	Globose	Dark green	MW143555	100
	TSAH	2.5 ± 0.05 × 2.3 ± 0.06	Globose	Dark green	MW143554	100

Note: final tested isolates were renamed after the submission of the ITS sequence to the GenBank database.

**Table 2:** LC_50_ values (conidia/mL) of the entomopathogenic fungi collected from rice fields against the fourth instar larvae of *Chilo suppressalis*.

Isolates	*N*	LC_50_ (Cl 95%) Conidia/mL	X^2^ (df)	Slope ± SE
BBAL1	150	2.1 × 10^4^ (1.1 × 10^3^ − 1.9 × 10^5^)	3.253 (3)	0.420 ± 0.068
BBAL2	150	2.3 × 10^5^ (4.7 × 10^4^ − 1.4 × 10^6^)	0.377 (3)	0.313 ± 0.059
BBAL3	150	5.6 × 10^4^ (1.1 × 10^4^ − 2.4 × 10^5^)	0.689 (3)	0.353 ± 0.062
BBAL4	150	9.6 × 10^4^ (1.9 × 10^4^ − 4.8 × 10^5^)	0.820 (3)	0.331 ± 0.060
BBBL1	150	1.5 × 10^5^ (3.1 × 10^4^ − 7.9 × 10^5^)	0.453 (3)	0.321 ± 0.059
BBBL2	150	3.9 × 10^5^ (7.2 × 10^4^ − 3.2 × 10^6^)	0.327 (3)	0.292 ± 0.058
BBLN1	150	1 × 10^4^ (2.9 × 10^3^ − 9.9 × 10^4^)	3.084 (3)	0.380 ± 0.065
BBNL2	150	5.4 × 10^4^ (1.1 × 10^4^ − 2.4 × 10^5^)	2.285 (3)	0.345 ± 0.061
BBLN3	150	1.5 × 10^5^ (4 × 10^4^ − 6.7 × 10^5^)	2.455 (3)	0.385 ± 0.064
BBLD1	150	1.1 × 10^5^ (2.3 × 10^4^ − 5.5 × 10^5^)	1.280 (3)	0.336 ± 0.060
BBLD2	150	1 × 10^5^ (2.6 × 10^4^ − 4.5 × 10^5^)	1.126 (3)	0.379 ± 0.063
BBLD3	150	9.5 × 10^4^ (2.3 × 10^4^ − 3.9 × 10^5^)	1.156 (3)	0.386 ± 0.064
BBLD4	150	1.2 × 10^5^ (2.7 × 10^4^ − 5.8 × 10^5^)	2.365 (3)	0.351 ± 0.061
BBLD5	150	4.4 × 10^5^ (9.9 × 10^4^ − 2.7 × 10^6^)	1.126 (3)	0.336 ± 0.061
BBRT1	150	4.9 × 10^5^ (6.8 × 10^4^ − 6.8 × 10^6^)	1.320 (3)	0.247 ± 0.056
BBRT2	150	3.4 × 10^5^ (7.8 × 10^4^ − 1.9 × 10^6^)	0.534 (3)	0.342 ± 0.061
BBRR1	150	2.2 × 10^4^ (4.6 × 10^3^ − 8.8 × 10^4^)	1.477 (3)	0.337 ± 0.064
BBRR2	150	2.4 × 10^5^ (6.1 × 10^4^ − 1.1 × 10^6^)	0.776 (3)	0.375 ± 0.063
BBSI	150	1.4 × 10^5^ (3.2 × 10^4^ − 7.6 × 10^5^)	0.513 (3)	0.337 ± 0.060
BBFN	150	2.3 × 10^5^ (4.5 × 10^4^ − 1.5 × 10^6^)	1.470 (3)	0.307 ± 0.059
BBAI	150	1.9 × 10^5^ (4.2 × 10^4^– 1 × 10^6^)	0.539 (3)	0.332 ± 0.060
HSAL	150	7.9 × 10^5^ (1.5×10^5^ − 7.3 × 10^6^)	1.985 (3)	0.309 ± 0.052
HSBL	150	1.6 × 10^6^ (2.4 × 10^5^ − 1.5 × 10^7^)	0.449 (3)	0.297 ± 0.059
MASA	150	7.1 × 10^4^ (1.6 × 10^4^ − 2.9 × 10^5^)	2.325 (3)	0.374 ± 0.062
MAAI	150	1.6 × 10^5^ (3.4 × 10^4^ − 9.4 × 10^5^)	1.743 (3)	0.325 ± 0.060
MAAL	150	3.6 × 10^5^ (7.4 × 10^4^ − 2.4 × 10^6^)	0.820 (3)	0.315 ± 0.059

Note: calculations were carried out by POLO-Plus software.

**Table 3:** LT_50_ values (days) of the entomopathogenic fungi collected from rice fields against the fourth instar larvae of *Chilo suppressalis*.

Isolates	LT_50_ (Cl 95%) Days	X^2^ (df)	Slope ± SE
BBAL1	3.45 (2.55–4.43)	11.948 (5)	4.007 ± 0.496
BBAL2	4.16 (3.16–5.21)	20.914 (8)	3.270 ± 0.347
BBAL3	3.87 (3.28–4.45)	9.303 (7)	4.354 ± 0.465
BBAL4	4.03 (3.47–4.55)	7.305 (8)	4.12 ± 0.447
BBBL1	4.18 (3.50–4.86)	12.719 (8)	3.983 ± 0.410
BBBL2	4.63 (4.13–5.13)	0.883 (8)	3.803 ± 0.419
BBLN1	3.15 (2.36–4)	13.895 (5)	3.302 ± 0.423
BBNL2	3.70 (2.96–4.45)	12.155 (7)	3.545 ± 0.392
BBLN3	3.75 (2.96–4.54)	14.866 (7)	3.899 ± 0.416
BBLD1	4.05 (3.25–4.84)	15.98 (8)	3.654 ± 0.376
BBLD2	4.28 (3.30–5.23)	27.697 (8)	3.654 ± 0.376
BBLD3	3.81 (3.38–4.21)	6.745 (7)	4.248 ± 0.456
BBLD4	3.66 (3.24–4.05)	5.112 (8)	4.166 ± 0.420
BBLD5	4.23 (3.74–2.4.71)	3.729 (8)	3.648 ± 0.386
BBRT1	4.91 (4.03–5.78)	15.756 (8)	3.623 ± 0.396
BBRT2	4.77 (4.29–5.24)	3.858 (8)	4.141 ± 0.445
BBRR1	2.71 (2.29–3.10)	5.961 (6)	3.260 ± 0.383
BBRR2	4.16 (3.52–4.78)	12.398 (8)	4.373 ± 0.441
BBSI	4.33 (3.77–4.87)	8.335 (8)	4.084 ± 0.419
BBFN	4.48 (3.88–5.08)	9.194 (8)	3.99 ± 0.414
BBAI	4.41 (3.67–5.14)	14.25 (8)	4.085 ± 0.419
HSAL	4.65 (3.83–5.54)	13.498 (7)	4.070 ± 0.457
HSBL	5.21 (4.72–5.74)	2.075 (7)	4.434 ± 0.526
MASA	3.69 (3.01–4.38)	9.280 (6)	4.021 ± 0.470
MAAI	4.91 (4.03–5.87)	17.220 (7)	3.723 ± 0.410
MAAL	4.14 (3.64–4.64)	1.887 (8)	3.386 ± 0.366

Note: calculations were carried out by POLO-Plus software.

**Table 4:** Amount of the hydrophobin (mg/mL) in the collected entomopathogenic fungi from the larvae of *Chilo suppressalis*.

Isolates	Amount of Hydrophobin (mg/mL)
ALRR	0.0603 ± 0.007 ^fghi^
ALFN	0.0628 ± 006 ^efghi^
ALAL	0.0535 ± 0.005 ^hi^
ALRT	0.0586 ± 0.002 ^ghi^
ALLN	0.0627 ± 0.003 ^efghi^
AMRT	0.0687 ± 0.003 ^cdefgh^
AMAI	0.0663 ± 0.005 ^cdefgh^
AMAL	0.0656 ± 0.001 ^defgh^
AMBL	0.0679 ± 0.002 ^cdefgh^
ASAI	0.038 ± 0.005 ^j^
BBAL1	0.0953 ± 0.001 ^a^
BBAL2	0.0745 ± 0.004 ^bcdefgh^
BBAL3	0.0765 ± 0.002 ^abcdefg^
BBAL4	0.0749 ± 0.002 ^bcdefgh^
BBBL1	0.0846 ± 0.002 ^abcd^
BBBL2	0.0780 ± 0.001 ^abcdefgh^
BBLN1	0.822 ± 0.003 ^abcde^
BBLN2	0.0803 ± 0.003 ^abcde^
BBLN3	0.0756 ± 0.003 ^abcdefgh^
BBLD1	0.0854 ± 0.004 ^abc^
BBLD2	0.0796 ± 0.006 ^abcdefg^
BBLD3	0.0704 ± 0.003 ^bcdefgh^
BBLD4	0.0782 ± 0.002 ^abcdefg^
BBLD5	0.0897 ± 0.002 ^ab^
BBRT1	0.0704 ± 0.004 ^bcdefgh^
BBRT2	0.0774 ± 0.002 ^abcdefg^
BBRR1	0.0767 ± 0.002 ^abcdefg^
BBRR2	0.0677 ± 0.003 ^cdefgh^
BBSI	0.0762 ± 0.001 ^abcdefg^
BBFN	0.0729 ± 0.001 ^bcdefgh^
BBAI	0.0832 ± 0.002 ^abcd^
HSAL	0.0816 ± 0.001 ^abcde^
HSBL	0.0684 ± 0.003 ^cdefgh^
MASA	0.0805 ± 0.002 ^abcde^
MAAI	0.0631 ± 0.002 ^ifghi^
MAAL	0.0714 ± 0.001 ^bcdefgh^
TSRT	0.0036 ± 0.002 ^j^
TSAH	0.043 ± 0.002 ^ij^

Note: Statistical differences are shown by different letters (Tukey’s test, *p* ≤ 0.05).

**Table 5:** Thermotolerance and cold activity of the entomopathogenic fungi collected from the larvae of *Chilo suppressalis*.

Isolates	Conidial Germination (%)			
	Exposure to 45 °C		Incubating at 4 °C	
	1 h	2 h	7 Day	14 Day
ALRR	29.86 ± 1.2 ^mnopq^	10.90 ± 0.6 ^op^	84.36 ± 1.6 ^ghijk^	90.53 ± 1.5 ^abcdef^
ALFN	27.78 ± 1 ^nopq^	12.21 ± 0.3 ^mnop^	94.20 ± 0.9 ^abcde^	95.85 ± 0.9 ^abcd^
ALAL	37.75 ± 2.1 ^jklmn^	16.39 ± 0.5 ^ijklmno^	88.58 ± 1. ^abcdefghij^	93.98 ± 0.9 ^abcde^
ALRT	29.54 ± 1.5 ^mnopq^	11.98 ± 0.9 ^nop^	95.66 ± 1.6 ^abc^	96.28 ± 0.8 ^abc^
ALLN	35.46 ± 1.3 ^klmnop^	12.98 ± 0.9 ^klmnop^	86.39 ± 1.7 ^efghij^	90.72 ± 0.9 ^abcdef^
AMRT	22.67 ± 1.6 ^pq^	9.07 ± 0.6 ^op^	91.14 ± 1.4 ^abcdefgh^	96.49 ± 1.3 ^abc^
AMAI	27.13 ± 1.8 ^pq^	10.64 ± 0.6 ^pq^	94.98 ± 0.7 ^abcd^	97.07 ± 0.9 ^ab^
AMAL	29.95 ± 1.9 ^mnopq^	12.76 ± 0.7 ^klmnop^	87.13 ± 1.6 ^defghij^	94.30 ± 0.9 ^abcde^
AMBL	21.91 ± 1.7 ^pq^	8.93 ± 0.5 ^pq^	95.95 ± 1.3 ^a^	98.08 ± 0.6 ^a^
ASAI	97.33 ± 0.8 ^a^	92.41 ± 0.8 ^a^	0 ^l^	0 ^g^
BBAL1	75.25 ± 1.6 ^bc^	38.55 ± 1.6 ^bcd^	93.19 ± 0.7 ^abcdef^	97.52 ± 0.7 ^ab^
BBAL2	57.24 ± 1.4 ^fg^	23.55 ± 1.1 ^gh^	92.14 ± 0.8 ^abcdefg^	94.42 ± 0.5 ^abcde^
BBAL3	38.14 ± 1.6 ^klm^	14.43 ± 0.9 ^klmnop^	88.45 ± 1.6 ^abcdefghij^	93.40 ± 0.9 ^abcde^
BBAL4	36.36 ± 1.9 ^jklmno^	12.60 ± 1.0 ^lmnop^	87.80 ± 1.0 ^cdefghij^	91.94 ± 0.7 ^abcdef^
BBBL1	50.41 ± 1.3 ^ghi^	18.93 ± 1.1 ^hijkl^	89.30 ± 1.1 ^abcdefghij^	94.65 ± 0.7 ^abcd^
BBBL2	51.03 ± 1.8 ^ghi^	20.95 ± 1.0 ^ghij^	87.96 ± 1.4 ^bcdefghij^	91.07 ± 1 ^abcdef^
BBLN1	76.29 ± 1.9 ^b^	41.58 ± 1.3 ^bc^	95.84 ± 0.7 ^ab^	97.29 ± 0.9 ^ab^
BBLN2	69.56 ± 1.2 ^bcd^	35.83 ± 1.8 ^cde^	86.54 ± 0.9 ^efghij^	92.33 ± 0.8 ^abcde^
BBLN3	42.47 ± 1.7 ^ijkl^	21.85 ± 1.2 ^ghi^	88.24 ± 1.6 ^abcdefghij^	93.19 ± 0.9 ^abcde^
BBLD1	43.06 ± 1.5 ^hijk^	26.70 ± 1.1 ^fg^	93.78 ± 0.9 ^abcdef^	91.51 ± 6.1 ^abcde^
BBLD2	57.79 ± 1.3 ^efg^	30.97 ± 1.1 ^ef^	86.48 ± 1.1 ^efghij^	91.68 ± 0.8 ^abcde^
BBLD3	26.51 ± 1.4 ^pq^	11.27 ± 0.7 ^op^	94.98 ± 0.9 ^abcd^	96.86 ± 0.7 ^ab^
BBLD4	37.00 ± 1.1 ^jklmno^	14.55 ± 1.1jkl ^mnop^	85.03 ± 1.1 ^fghijk^	90.64 ± 0.9 ^abcdef^
BBLD5	33.88 ± 2.3 ^lmnop^	12.19 ± 1.1 ^mnop^	85.95 ± 1.2 ^fghijk^	91.11 ± 1 ^abcdef^
BBRT1	51.95 ± 1.4 ^gh^	19.08 ± 1.1 ^hijk^	93.83 ± 0.9 ^abcdef^	96.50 ± 0.7 ^abc^
BBRT2	35.18 ± 1.7 ^klmnop^	18.59 ± 0.8 ^hijklm^	90.53 ± 0.8 ^abcdefghi^	95.47 ± 0.8 ^abcd^
BBRR1	72.63 ± 1.2 ^bc^	33.95 ± 1.6 ^de^	94.23 ± 0.9 ^abcde^	98.14 ± 0.5 ^a^
BBRR2	28.27 ± 1.2 ^opq^	12.29 ± 1.6 ^mnop^	84.83 ± 1.2 ^fghijk^	93.03 ± 0.9 ^abcde^
BBSI	42.16 ± 1.7 ^ijkl^	18.04 ± 1.1 ^hijklmn^	82.78 ± 1.3 ^ijk^	90.66 ± 1.2 ^abcdef^
BBFN	44.60 ± 2.1 ^hij^	19.08 ± 1.1 ^hijk^	87.55 ± 1.2 ^defghij^	91.90 ± 1.2 ^abcde^
BBAI	42.76 ± 2.3 ^ijkl^	18.59 ± 0.8 ^hijklm^	87.60 ± 1.4 ^defghij^	93.38 ± 1.1 ^abcde^
HSAL	11.38 ± 0.9 ^r^	2.90 ± 0.7 ^q^	83.48 ± 1.6 ^hijk^	88.83 ± 1.5 ^cdef^
HSBL	9.77 ± 0.6 ^r^	1.59 ± 0.5 ^q^	82.95 ± 3.3 ^ijk^	86.87 ± 1.4 ^ef^
MASA	73.41 ± 1.2 ^bc^	36.91 ± 2.1 ^bcde^	89.87 ± 2.1 ^abcdefgij^	94.93 ± 1.7 ^abcd^
MAAI	67.65 ± 1.6 ^bcd^	33.82 ± 1.5 ^de^	83.93 ± 1.4 ^hijk^	89.85 ± 1.3 ^bcdef^
MAAL	63.04 ± 1.4 ^def^	31.31 ± 1.2 ^ef^	82.04 ± 1.5 ^jk^	88.30 ± 0.7 ^def^
TSRT	69.8 ± 1.7 ^bcd^	40.57 ± 1.7 ^bc^	88.11 ± 1.2 ^abcdefghij^	93.64 ± 0.8 ^abcde^
TSAH	75.05 ± 1.7 ^bc^	43.29 ± 1.2 ^b^	84.74 ± 2.3 ^ghijk^	89.89 ± 0.8 ^bcdef^

Note: Statistical differences are shown by different letters.
